# Estimation and model selection for finite mixtures of Tukey’s *g*- &-*h* distributions

**DOI:** 10.1007/s11222-025-10596-9

**Published:** 2025-03-15

**Authors:** Tingting Zhan, Misung Yi, Amy R. Peck, Hallgeir Rui, Inna Chervoneva

**Affiliations:** 1 Division of Biostatistics and Bioinformatics, Department of Pharmacology, Physiology and Cancer Biology, Thomas Jefferson University, 130 S. 9th Street, 17th Floor, Philadelphia, PA 19107 USA; 2 Department of Statistics and Data Science, College of Software Convergence, Dankook University, Yongin-si, Gyeonggi-do Republic of Korea; 3 Division of Cancer Biology, Department of Pharmacology, Physiology and Cancer Biology, Thomas Jefferson University, Philadelphia, USA

**Keywords:** Finite mixtures, Tukey’s *g*- &-*h* distribution, Indirect estimator, Quantile least Mahalanobis distance, Cellular protein level

## Abstract

A finite mixture of distributions is a popular statistical model, which is especially meaningful when the population of interest may include distinct subpopulations. This work is motivated by analysis of protein expression levels quantified using immunofluorescence immunohistochemistry assays of human tissues. The distributions of cellular protein expression levels in a tissue often exhibit multimodality, skewness and heavy tails, but there is a substantial variability between distributions in different tissues from different subjects, while some of these mixture distributions include components consistent with the assumption of a normal distribution. To accommodate such diversity, we propose a mixture of 4-parameter Tukey’s *g*- &-*h* distributions for fitting finite mixtures with both Gaussian and non-Gaussian components. Tukey’s *g*- &-*h* distribution is a flexible model that allows variable degree of skewness and kurtosis in mixture components, including normal distribution as a particular case. Since the likelihood of the Tukey’s *g*- &-*h* mixtures does not have a closed analytical form, we propose a quantile least Mahalanobis distance (QLMD) estimator for parameters of such mixtures. QLMD is an indirect estimator minimizing the Mahalanobis distance between the sample and model-based quantiles, and its asymptotic properties follow from the general theory of indirect estimation. We have developed a stepwise algorithm to select a parsimonious Tukey’s *g*- &-*h* mixture model and implemented all proposed methods in the R package QuantileGH available on CRAN. A simulation study was conducted to evaluate performance of the Tukey’s *g*- &-*h* mixtures and compare to performance of mixtures of skew-normal or skew-*t* distributions. The Tukey’s *g*- &-*h* mixtures were applied to model cellular expressions of Cyclin D1 protein in breast cancer tissues, and resulting parameter estimates evaluated as predictors of progression-free survival.

## Introduction

Finite mixtures are popular distribution models with a continuously expanding range of applications. Data exhibiting multimodality are quite common in biomedical sciences, and finite mixtures are especially useful for modeling distinct subpopulations with some overlap and clustering (Titterington et al. 1985; McLachlan and Basford 1988; Everitt and Hand 1981; McLachlan et al. 2019). The finite mixture of Gaussian/normal components most commonly used to identify heterogeneous subpopulations as components of the mixture (Reynolds 2009; Geoffrey 2000, 2014; Benaglia et al. 2009), including the application to assignment of cell identity from single-cell multiplexed imaging and proteomic data (Geuenich et al. 2021).

**Fig. 1:**
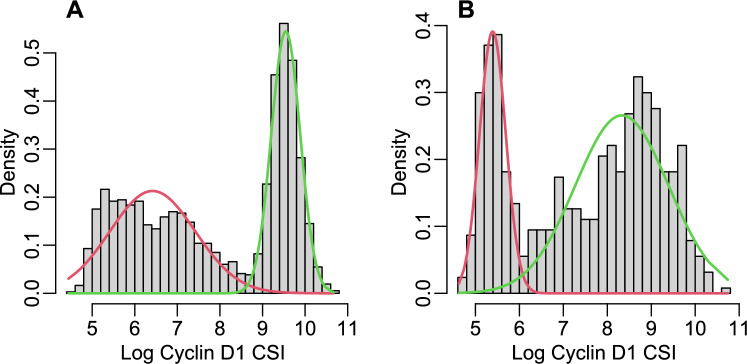
Examples of fitting 2-component normal mixtures to Cyclin D1 Log CSI expression levels with poor fit of the normal distribution in the first component **A** or in the second component **B**

However, the distributions of mixture components may be skewed and have heavy tails in many applications, especially biomedical. For instance, such distributions are often observed for the data generated by quantification of cellular signal intensity (CSI) of protein expression in the immunofluorescence immunohistochemistry (IF-IHC) images. Figure 1 shows histograms of CSI distributions of Cyclin D1 protein quantified using IF-IHC technology in a tissue microarray (TMA) cores of breast cancer patients. For many other proteins heterogeneously expressed in tumor tissue, the distributions of CSI levels are often bimodal and heavily skewed like the ones shown in Fig. 1. It is biologically meaningful to assume that such CSI distributions are mixtures of a lower component representing the background noise and an upper component(s) representing the actual signal, but normal distribution is not appropriate in many cases. For example, see the first component in Fig. 1A and for the second component in Fig. 1B.

In this work, we develop a finite mixture of Tukey’s *g*- &-*h* distributions to accommodate components with variable degrees of skewness and kurtosis. The normal distribution family can be expanded to include heavy tailed and skewed distributions within the *g*- &-*h* distributional family introduced by Tukey (Tukey 1977). This family represents a rich class of common univariate distributions with different levels of skewness and elongation. Tukey’s *g*- &-*h* distributions can approximate a wide variety of commonly used distributional shapes, such as normal, lognormal, Weibull, exponential and many other distributions that do not have finite first four moments, such as the Cauchy distribution (Martinez and Iglewicz 1984). Mixtures of two Tukey’s *g*-distributions, a subclass of Tukey’s *g*- &-*h* distributions, were recently proposed for econometric applications (Vitiello 2008; Jiménez and Arunachalam 2016), but Tukey’s *g*- &-*h* mixtures that include *h*-parameter have not been considered in the literature before. Since Tukey’s *g*- &-*h* family includes subclasses of normal distributions, *g*-distributions, and *h*-distributions, using Tukey’s *g*- &-*h* mixtures allows different components to have different number of parameters. That is one component may have a normal distribution, while the other may have Tukey’s *g*- &-*h* distribution with 4 parameters.

The likelihood of a single Tukey’s *g*- &-*h* distribution and Tukey’s *g*- &-*h* mixture do not have a closed analytical form, and the maximum likelihood estimation is not feasible. Therefore, we propose a Quantile Least Mahalanobis Distance (QLMD) estimator of Tukey’s *g*- &-*h* mixture parameters. This QLMD estimator minimizes the Mahalanobis distance between selected empirical sample quantiles and corresponding model-based quantiles. The QLMD estimator may be viewed as an indirect estimator and its consistency and asymptotic normality follows from the general theory of indirect estimation (Gourieroux et al. 1993; Gouriéroux et al. 1996). This estimator generalizes the quantile based least squares (QLS) estimator (Xu et al. 2014) by accommodating a mixture rather than a single Tukey’s *g*- &-*h* distribution and incorporating the covariance matrix of sample quantiles in the objective function. The QLMD estimator can be made robust to extreme outliers if the quantiles selected as ancillary parameters exclude the extreme tail probabilities, similar to the QLS estimator (Xu et al. 2014).

To facilitate selection of a parsimonious Tukey’s *g*- &-*h* mixture model, we developed an algorithm which combines forward selection of *g*- &-*h* parameters for a given number of components and forward selection of the number of components to maximize a penalized likelihood. All methods developed were implemented in the R (R Core Team 2022) package QuantileGH, available at https://CRAN.R-project.org/package=QuantileGH.

Previously proposed finite mixtures of continuous non-Gaussian parametric distributions include mixtures of *t*-distributions (Peel and McLachlan 2000), skew-normal distributions (Lin et al. 2007; Basso et al. 2010), multivariate skew *t*-distributions (Lin 2010; Lee and McLachlan 2014), skew elliptical distributions, including the scale mixtures of the skew-normal distribution (Branco and Dey 2001; Genton 2004; Prates et al. 2013), Gamma distributions (Wiper 2001; Young et al. 2019), and generalized hyperbolic distributions (Browne and McNicholas 2015). Finite scale mixtures of skew-normal distributions have been also used to model random errors in the context of robust mixture regression (Zeller et al. 2016).

The scale mixtures of skew-*t* distributions have the same number of parameters as the proposed full/unconstrained Tukey’s *g*- &-*h* mixture model, and both models incorporate parameters for skewness and kurtosis. The main advantage of our model is flexibility with respect to combining components with different complexity and required number of parameters. That is, a parsimonious Tukey’s *g*- &-*h* mixture model may include components with normal distribution combined with Tukey’s *g*-distribution or *h*-distribution or *g*- &-*h* distribution. This allows adaptive complexity of the model potentially improving interpretability.

A simulation study was conducted to (i) evaluate the finite sample performance of the QLMD estimator with and without model selection for estimating parameters of 2-component Tukey’s *g*- &-*h* mixture models and (ii) compare fitted 2-component mixtures with Tukey’s *g*- &-*h*, skew-normal, and skew-*t* components. The simulation results indicate that Tukey *g*- &-*h* mixtures provide more accurate estimates of component means that are more robust to mis-specification of the component distribution.

We then applied Tukey’s *g*- &-*h* mixture to modeling distributions of cell-level Cyclin D1 protein expression in breast cancer tissues. Full or reduced 2-component mixtures as well as the models with the optimal number of components were estimated for distribution of Cyclin D1 expression in cells from each tissue. The fitted models were used to estimate the location parameter of the upper end component, which was evaluated as predictor of progression free survival (PFS) in breast cancer patients. The best prognostic value was achieved using the parsimonious Tukey’s *g*- &-*h* mixture. This highlights the advantage of the proposed parsimonious Tukey’s *g*- &-*h* mixture model that allows optimizing the complexity of the components in the mixture. We have also compared the goodness of fit for optimal Tukey’s *g*- &-*h*, skew-normal, skew-*t*, and normal mixtures applied to Cyclin D1 expression in each tissue. The optimal Tukey’s *g*- &-*h* mixtures had similar goodness of fit, but smaller number of parameters for the majority of breast cancer tissues, as compared to alternative optimal non-Gaussian mixtures.

## Finite mixtures of Tukey’s *g*- &-*h* distributions

### Tukey’s *g*- &-*h* distributions

Tukey’s *g*- &-*h* random variable $T_{(A,B,g,h)}$ (Tukey 1977) is defined through a monotone transformation of the standard normal variable *Z* (Hoaglin 1985),

$$\begin{aligned}&T_{(A,B,g,h)}\\&\quad ={\left\{ \begin{array}{ll} A + B\cdot G(Z)\cdot Z & g\ne 0,\ g\text {-distribution} \\ A + B\cdot H(Z)\cdot Z & h>0,\ h\text {-distribution} \\ A + B\cdot G(Z)\cdot H(Z)\cdot Z & g\ne 0,h>0,\ gh\text {-distribution} \\ \end{array}\right. } \end{aligned}$$

where *A* is the location and $B>0$ is the scale parameter. The skewness is introduced by $G(z)=(e^{gz}-1)/gz$ with $G_0(z)=\lim _{g\rightarrow 0} G_{g \ne 0}(z)=1$. The kurtosis is introduced by $H(z)=e^{hz^2/2}$, $h\ge 0$. When $g=0$ and $h=0$ then $T_{(A,B,g,h)} = A + BZ$ follows a normal distribution $N(A, B^2)$.

The quantile function of $T_{(A,B,g,h)}$ is

1 $$\begin{aligned} t_{(A, B, g, h)}(p)={\left\{ \begin{array}{ll} A+B z_p G(z_p) & g\ne 0 \\ A+B z_p H(z_p) & h>0 \\ A+B z_p G(z_p) H(z_p) & g\ne 0,\ h>0 \end{array}\right. } \end{aligned}$$

where $z_p$, $0<p<1$, is the *p*-th quantile of the standard normal distribution. The distribution function $F_{(A,B,g,h)}(t)=\text {Pr}(T_{(A, B, g, h)} \le t)=\text {Pr}(Z \le \zeta _{(A, B, g, h)}(t))$, where

2 $$\begin{aligned}&\zeta _{(A, B, g, h)}(t) \nonumber \\&\quad = {\left\{ \begin{array}{ll} g^{-1}\ln \big (gB^{-1}(t-A)+1\big ), & g\ne 0 \\ z:\ z H(z) = B^{-1}(t-A), & h>0 \\ z:\ z G(z) H(z) = B^{-1}(t-A), & g\ne 0,\ h>0 \end{array}\right. } \end{aligned}$$

The distribution function $F_{(A,B,g,h)}(t)$, as well as the inverse Tukey transformation $\zeta _{(A, B, g, h)}(t)$, do not have a closed analytical form when $h>0$, but their numerical values could be found by using a root-finding algorithm (Brent 1971). The density function $f_{(A,B,g,h)}(t)$ has a closed analytical form in terms of $\zeta _{(A, B, g, h)}$,

$$\begin{aligned} f_{(A,B,g,h)}(t) = \dfrac{e^{-z^2/2}}{\sqrt{2\pi }\cdot \partial t_{(A, B, g, h)}(z)/\partial z}\Bigg |_{z=\zeta _{(A, B, g, h)}(t)} \end{aligned}$$

where

$$\begin{aligned}&\dfrac{\partial t_{(A, B, g, h)}}{\partial z}\\&\quad = {\left\{ \begin{array}{ll} Be^{gz}, & g\ne 0\\ Be^{hz^2/2}(1+hz^2), & h>0 \\ Be^{hz^2/2}\left( e^{gz}+g^{-1}hz(e^{gz}-1)\right) , & g\ne 0,\ h>0 \end{array}\right. } \end{aligned}$$

### Finite mixture of Tukey’s *g*- &-*h* distributions

Consider the random variable $\textbf{T}_\phi$ of a *K*-component Tukey’s *g*- &-*h* mixture distribution,

3 $$\begin{aligned} \text {Pr}\left( \textbf{T}_\phi< t\right) = \sum ^K_{k=1} w_k\text {Pr}\left( T_{A_k,B_k,g_k,h_k}<t\right) \end{aligned}$$

The 5*K*-dimensional parameter vector $\phi = \{A_k, B_k, g_k, h_k, w_k: k = 1,\cdots ,K\}$ is a subject to constraint $\sum _k w_k = 1$, the ordered location constraint $A_1< A_2< \cdots < A_K$, and the component-wise constraints $B_k>0$, $h_k\ge 0$, $k = 1,\cdots ,K$. To avoid non-identifiability due to empty or equal components and relabeling the components, we reparammeterize model (3) using $\varvec{\theta }$ in the unconstrained parameter space $\textbf{R}^{5K - 1}$,

4 $$\begin{aligned} \varvec{\theta }=&(A_1,d_2,\cdots ,d_k,\ \ b_1,\cdots , b_k,\ \ g_1,\cdots ,g_k,\nonumber \\ &\qquad \eta _1,\cdots ,\eta _k,\ \ \pi _2,\cdots ,\pi _K)^t \end{aligned}$$

where $d_k = \log (A_k - A_{k-1})$ and $\pi _k = \ln w_k - \ln w_1$ for $k = 2,\cdots ,K$; and $b_k = \log B_k$ and $\eta _k = \log h_k$ for $k = 1,\cdots ,K$. The use of $A_1$ and $d_k$ instead of all $A_k$, and multinomial logits $\pi _k$ instead of component weights $w_k$, guarantees formal identifiability of (3) (Frühwirth-Schnatter 1981) by ensuring that (3) is a mixture of *K* distinct (with different and ordered means) and nonempty components. The proof of generic identifiability (Teicher 1963) of a finite mixture of Tukey’s *g*- &-*h* distributions is given in Appendix A.

The unconstrained parameterization (4) does not allow $h_k=0$, which will be addressed later in Sect. 3.1. In further developments, let $\textbf{T}_\theta$ be the finite Tukey’s *g*- &-*h* mixture with unconstrained parameters $\varvec{\theta }$.

### Quantile least Mahalanobis distance (QLMD) estimator for finite Tukey’s *g*- &-*h* mixture

Given a random sample $X=(x_1, \cdots , x_n)^t$, the quantile least Mahalanobis distance (QLMD) estimator $\hat{\varvec{\theta }}_{\text {QLMD}}$ minimizes the Mahalanobis distance between the sample quantiles $\textbf{t} = (t_{p_1}, \cdots , t_{p_l})^t$ and the true quantiles $\textbf{t}_{\varvec{\theta }} = (t_{\varvec{\theta },p_1}, \cdots , t_{\varvec{\theta },p_l})^t$, for *l* pre-specified probabilities $0<p_1<\cdots<p_l<1$,

5 $$\begin{aligned} \hat{\varvec{\theta }}_\text {QLMD} = \arg \min _{\varvec{\theta }} \left( \textbf{t} - \textbf{t}_{\varvec{\theta }}\right) ^t \hat{\textbf{V}}^{-1}_\textbf{t}\left( \textbf{t} - \textbf{t}_{\varvec{\theta }}\right) \end{aligned}$$

where $\hat{\textbf{V}}_{\textbf{t}}$ is a nonparametricly estimated covariance matrix of sample quantiles (Mosteller 1946) with the *ij*-th element

$$\begin{aligned} \left[ \hat{\textbf{V}}_\textbf{t}\right] _{ij} = \dfrac{\min \{p_i,p_j\}\ (1-\max \{p_i,p_j\})}{\hat{f}(t_{p_i}) \hat{f}(t_{p_j})}, \quad i,j = 1,\cdots , l \end{aligned}$$

and kernel density estimator $\hat{f}$. Under the assumptions listed in Appendix B, the indirect estimation theory (Gourieroux et al. 1993; Gouriéroux et al. 1996) implies that $\hat{\varvec{\theta }}_{\text {QLMD}}$ is $\sqrt{n}$-consistent for estimating the true parameter $\varvec{\theta }_0$ and, as $n\rightarrow \infty$,

6 $$\begin{aligned}&\sqrt{n}\left( \hat{\varvec{\theta }}_{\text {QLMD}} - \varvec{\theta }_0\right) \nonumber \\&\qquad {\mathop {\rightarrow }\limits ^{D}} \text {N}\left( \textbf{0}, \left( \textbf{J}_{\textbf{t}_{\varvec{\theta }_0}}^t\textbf{J}_{\textbf{t}_{\varvec{\theta }_0}}\right) ^{-1}\textbf{J}_{\textbf{t}_{\varvec{\theta }_0}}^t \textbf{V}_{\textbf{t}_{\varvec{\theta }_0}} \textbf{J}_{\textbf{t}_{\varvec{\theta }_0}}\left( \textbf{J}_{\textbf{t}_{\varvec{\theta }_0}}^t\textbf{J}_{\textbf{t}_{\varvec{\theta }_0}}\right) ^{-1}\right) \end{aligned}$$

where $\textbf{J}_{\textbf{t}_{\varvec{\theta }_0}}$ is the $l \times (5K-1)$ Jacobian matrix with the *ij*-th partial derivative

$$\begin{aligned} \left[ \textbf{J}_{\textbf{t}_{\varvec{\theta }_0}}\right] _{ij} = \dfrac{\partial t_{\varvec{\theta },p_i}}{\partial \theta _j}\Bigg |_{\varvec{\theta } = \varvec{\theta }_0}\qquad i = 1,\cdots ,l \ \text {and}\ j = 1,\cdots ,5K-1 \end{aligned}$$

and matrix $\textbf{V}_{\mathbf {t_{\varvec{\theta }_0}}}$ is the asymptotic covariance matrix of sample quantiles of Tukey’s *g*- &-*h* mixture with the true parameter vector $\varvec{\theta }_0$. Matrix $\textbf{V}_{\mathbf {t_{\varvec{\theta }_0}}}$, additional assumptions needed for consistency and asymptotic normality of $\hat{\varvec{\theta }}_{\text {QLMD}}$, and sketch of the proof are given in Appendix B. The approximate asymptotic covariance matrix of the original parameter $\phi$ can be obtained from the asymptotic covariance (6) of the unconstrained parameter $\varvec{\theta }$ in (4) using the delta method, see detail in Appendix B.

### Implementation of QLMD estimator

**Fig. 2:**
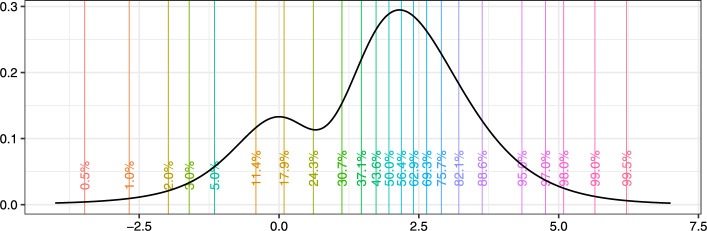
Selection of *p*

The number of quantiles *l* for QLMD estimation has to be at least as large as the number of parameters in the mixture to satisfy the assumptions listed in Appendix B. Quantiles corresponding to equidistant probabilities may be considered a natural choice, but our numeric studies suggest that inclusion of additional quantiles at the tails improves the accuracy of *g* and *h* parameter estimation. For mixtures with 2–3 components, we recommend using fifteen (15) equidistant probabilities between .05 and .95, and denser probabilities in each tail of the distribution, .005, .01, .02, .03, .97, .98, .99, .995. Figure 2 illusttrates this selection of percentages on a two-component Tukey’s *g*- &-*h* mixtures with parameters $(A_1,A_2)=(0,2.5)$, $B_1=B_2=1$, $(g_1,g_2)=(0,.3)$, $(h_1,h_2)=(.2,0)$ and $(w_1,w_2)=(.33,.67)$.

Since QLMD estimator is potentially sensitive to the initial parameter values, we propose to use two different approaches for computing the starting values and then select the starting values that provide a better fit for the data.

The first approach is to use the normal mixture estimates (R package mixtools, Benaglia et al. (2009)) as starting values for means, standard deviations and mixing proportions and let $g=h=0$ for all components.

For the second approach, we first apply *k*-means clustering (Cuesta-Albertos et al. 1997) to trimmed data with $5\%$ of the observations being trimmed. Then the Mahalanobis distance from each trimmed observation to each of the *K* clusters is computed. Each trimmed observation is assigned to the closest cluster in terms of Mahalanobis distance. This approach of trimmed *k*-means clustering with re-assignment, is superior to the *k*-means clustering (Hartigan and Wong 1979), which could identify the long tails due to heavy skewedness and/or kurtosis as standalone cluster(s). The relative proportions of the resulting re-assigned clusters are used as initial estimates of the mixing parameters *w*’s. The cluster-wise Tukey’s *g*- &-*h* parameter estimates are computed using a slightly modified letter-value based estimates of Hoaglin (1985) (see details in Appendix C). We use letter-value based estimates based on lower half-spread (LHS) to estimate the distribution parameters of the cluster with the smallest location parameter, upper half-spread (UHS) for the cluster with the largest location parameter, and full-spread for the rest of the clusters.

In the simulation studies, we observed that the second approach yields better starting values for Tukey’s *g*- &-*h* mixtures with non-trivial *g* and/or *h* parameters, while the first approach may yield better starting values in cases when many *g* and/or *h* parameters are zero. Therefore, we obtain both types of initial estimates and choose the one with greater numerically approximated likelihood as the starting values. The QLMD of $\varvec{\theta }$ in (4) is then obtained using Nelder-Mead minimization (Nelder and Mead 1965).

## Parsimonious model selection

We developed two forward selection algorithms to find a parsimonious *K*-component Tukey’s mixture with some or all $g_k=0$ and/or $h_k=0$, and to find an optimal number of components no greater than a user-specified $K_\text {max}$.

### *K*-component *gh*-parsimonious mixture

Let $\varvec{\rho }= (g_1,\cdots ,g_K,h_1,\cdots ,h_K)^t$ be a sub-vector of *g*- &-*h* parameters of a *K*-component Tukey’s *g*- &-*h* mixture distribution. We call the mixture *gh*-*constrained* if $g_k=0$ or $h_k=0$, for at least one $k=1,\cdots ,K$. If $g_k=h_k=0$ for all $k=1,\cdots ,K$, i.e., $\varvec{\rho }={\textbf {0}}$, the mixture reduces to a normal mixture.

The forward stepwise selection starts with a *K*-component normal mixture and adds a non-zero *g* or *h* parameter only if this increases the penalized log-likelihood $\text {PL}(\theta )$ with the same penalty as in Akaike (AIC, Akaike 1974) or Bayesian information criterion (BIC, Schwarz 1978),

$$\begin{aligned} \text {PLa}(\theta )&= \ln \mathcal {L}(\theta ) - r&\text {AIC-like} \\ \text {PLb}(\theta )&= \ln \mathcal {L}(\theta ) - r\ln (n)/2&\text {BIC-like} \end{aligned}$$

where *r* is the number of parameters and *n* is the sample size. The likelihood $\mathcal {L}(\theta )$ has a closed form in terms of numerical solution to equation (2).

The algorithm proceeds as following:

1. Compute QLMD estimate $\hat{\varvec{\theta }} ^0$ with all $g_k=h_k=0$, $k=1,\cdots ,K$, i.e. a normal mixture estimate.
2. For each index $m=1,\cdots ,2K$ of parameter sub-vector $\varvec{\rho }$, compute QLMD estimate $\hat{\varvec{\theta }} ^0_{+m}$ for a *gh*-constrained mixture with $\varvec{\rho } ^0_{+m} = (0,\cdots ,0,\rho _m,0,\cdots ,0)^t$. Here, $\varvec{\rho } ^0_{+m}$ can be a *g* or *h* parameter.
3. If $\text {PL}(\hat{\varvec{\theta }} ^0) > \text {PL}(\hat{\varvec{\theta }} ^0_{+m})$, for all $m = 1,\cdots ,2K$, then the normal mixture $\hat{\varvec{\theta }} ^0$ is *gh*-*parsimonious* and the algorithm is completed.
  Otherwise, let $b = \arg \max _{m}\text {PL}(\hat{\varvec{\theta }} ^0_{+m})$, denote $\hat{\varvec{\theta }} ^\text {new}=\hat{\varvec{\theta }} ^0_{+b}$, $\varvec{\rho }^\text {new} = \varvec{\rho } ^0_{+b}$, and proceed to the next step.
4. Let $\hat{\varvec{\theta }} ^c=\hat{\varvec{\theta }} ^\text {new}$, $\varvec{\rho }^c= \varvec{\rho }^\text {new}$ and $M^c$ be the set of indexes of zeros in $\varvec{\rho }^c$. If $M^c$ is empty, then $\hat{\varvec{\theta }} ^c$ is *gh*-parsimonious and the algorithm is completed. Otherwise, for each $m \in M^c$, compute QLMD estimate $\hat{\varvec{\theta }} ^c_{+m}$ with $\varvec{\rho } ^c_{+m} =\varvec{\rho }^c+(0,\cdots ,0,\rho _{m},0,\cdots ,0)^t$.
5. If $\text {PL}(\hat{\varvec{\theta }} ^c) > \text {PL}(\hat{\varvec{\theta }} ^c_{+m})$ for all $m \in M^c$, then $\hat{\varvec{\theta }} ^c$ is *gh*-parsimonious and the algorithm is completed.
  Otherwise, let $b = \arg \max _{m}\text {PL}(\hat{\varvec{\theta }} ^c_{+m})$ and denote $\hat{\varvec{\theta }} ^\text {new}=\hat{\varvec{\theta }} ^c_{+b}$, $\varvec{\rho }^\text {new} = \varvec{\rho } ^c_{+b}$ and return to Step 4.

The resulting *K*-component *gh*-parsimonious mixture can be either a full Tukey’s *g*- &-*h* mixture, a *gh*-constrained mixture, or simply a normal mixture.

### Optimal number of components

We select a *gh*-parsimonious mixture with the number of components ranging from $K=1$ to a user-specified $K_\text {max}$, in case of no *a priori* knowledge or preference for *K*. The forward selection algorithm proceeds as following:

1. Let $$K^c=1$$.
2. Let $\hat{\varvec{\theta }}_{K^c}$ and $\hat{\varvec{\theta }}_{K^c+1}$ be the QLMD estimates of *gh*-parsimonious mixtures described in Sect. 3.1, with $K^c$ and $K^c+1$ components, respectively.
3. If $\text {PL}(\hat{\varvec{\theta }}_{K^c}) \ge \text {PL}(\hat{\varvec{\theta }}_{K^c+1})$, then $\hat{\varvec{\theta }}_{K^c}$ is optimal and the selection is completed.
4. If $\text {PL}(\hat{\varvec{\theta }}_{K^c}) < \text {PL}(\hat{\varvec{\theta }}_{K^c+1})$ and $K^c+1 < K_\text {max}$, then let $K^\text {new} = K^c + 1$ and repeat Step 2 with $K^c=K^\text {new}$.
5. If $\text {PL}(\hat{\varvec{\theta }}_{K^c}) < \text {PL}(\hat{\varvec{\theta }}_{K^c+1})$ and $K^c+1 = K_\text {max}$, then $\hat{\varvec{\theta }}_{K^c+1}$, or $\hat{\varvec{\theta }}_{K_\text {max}}$ is optimal within the user-specified range of *K* and the selection is completed.

## Simulation studies

### Design

**Fig. 3:**
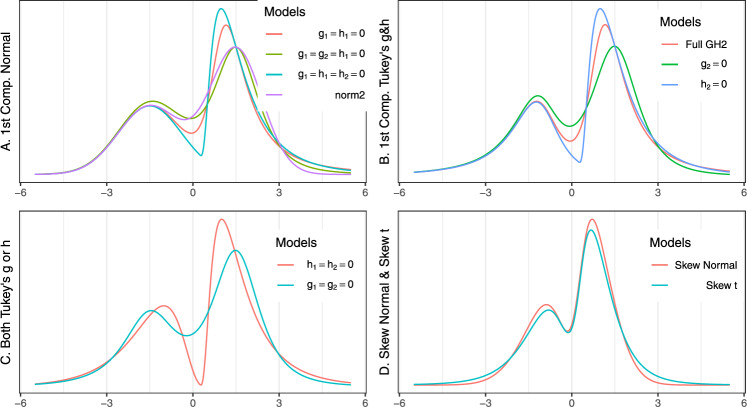
Simulated: Mixtures of Tukey’s *g*- &-*h*, skew normal and skew *t*

Random samples were generated from nine 2-component Tukey’s *g*- &-*h* mixtures (Table 1) with shared parameters $(A_1,A_2) = (-1.5,1.5)$, $(B_1,B_2)=(1.1,.9)$ and $(w_1,w_2)=(.4,.6)$ and skew-normal and skew-*t* (Basso et al. 2010) mixtures with parameters $(\xi _1,\xi _2) = (-.3,.3)$, $(\omega _1,\omega _2) = (1.1,.9)$, $(\alpha _1,\alpha _2) = c(-2, 3)$ and $\nu _1=\nu _2=5$. Figure 3 shows the density curves of all simulation scenarios. Five hundred (500) data sets were simulated for each simulation scenario with sample sizes $n=100$, 200, 500, 1000, 2000 and 5000.

**Table 1:** Simulated: Tukey’s *g*- &-*h* mixtures

Scenario	$g_1$	$g_2$	$h_1$	$h_2$
$g_{1}=h_{1}=0$	0	0.7	0	0.2
$g_{1}=g_{2}=h_{1}=0$	0	0	0	0.2
$g_{1}=h_{1}=h_{2}=0$	0	0.7	0	0
2-comp. Normal	0	0	0	0
Full 2-comp. Tukey *g*- &-*h*	$-$0.5	0.7	0.3	0.2
$g_{2}=0$	$-$0.5	0	0.3	0.2
$h_{2}=0$	$-$0.5	0.7	0.3	0
$h_{1}=h_{2}=0$	$-$0.5	0.7	0	0
$g_{1}=g_{2}=0$	0	0	0.3	0.2

The following mixture models were fitted,

- **Unconstrained**, estimating (i) $(\hat{\xi }_1,\hat{\xi }_2,\hat{\omega }_1,\hat{\omega }_2,\hat{\alpha }_1,\hat{\alpha }_2,\hat{w}_2)$ of skew-normal mixture model via function smsn.mix of R package mixsmsn (Prates et al. 2013) with option family = "Skew.normal"; (ii) $(\hat{\xi }_1,\hat{\xi }_2,\hat{\omega }_1,\hat{\omega }_2,\hat{\alpha }_1,\hat{\alpha }_2,\hat{\nu }_1,\hat{w}_2)$ of skew-*t* mixture model via function smsn.mix with option family = "Skew.t"; (iii) $(\hat{A}_1, \hat{A}_2, \hat{B}_1, \hat{B}_2, \hat{g}_1, \hat{g}_2, \hat{h}_1, \hat{h}_2, \hat{w}_2)$ for Tukey’s *g*- &-*h* mixture model using the QLMD estimator.
- **Known-Constraint**, estimating non-zero parameters listed in Table 1 using QLMD estimator and assuming that true constraints are known (for Tukey’s *g*- &-*h* mixture model only);
- *gh***-parsimonious** Tukey’s *gh*-parsimonious mixture model described in Sect. 3.1.

### Performance of QLMD estimator

**Fig. 4:**
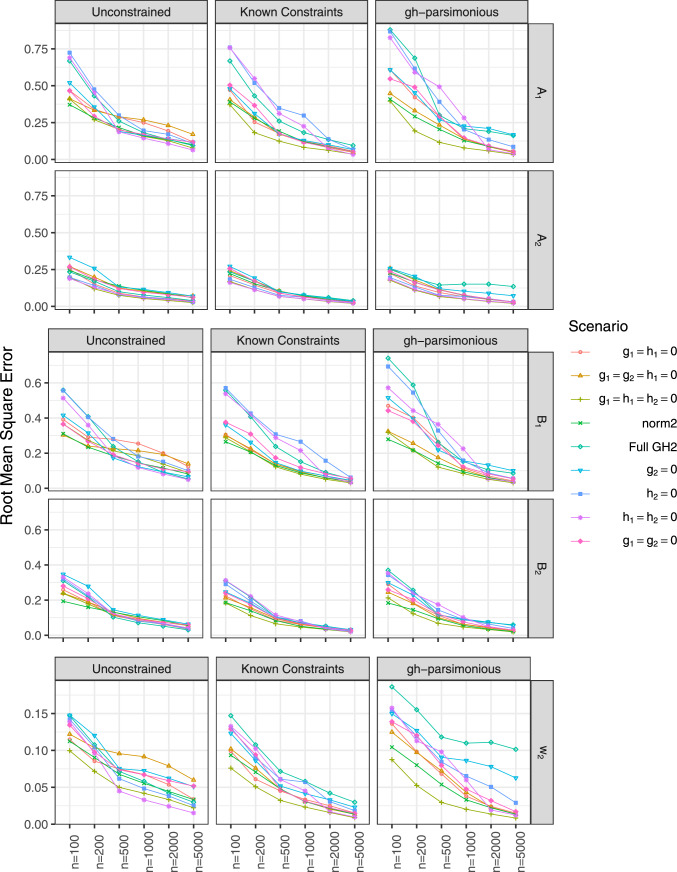
RMSE of the location parameter estimates ($A_1$, $A_2$), scale parameter estimates ($B_1$, $B_2$), and mixing proportion estimates ($w_2$)

Figure 4 presents the root mean squared errors (RMSEs) of location and scale parameters and mixing proportions estimates for Tukey’s *g*- &-*h* mixtures with all three estimation approaches. For applications, these parameters are usually of practical interest and can be compared to parameters of fitted normal mixtures. For all estimation approaches, all RMSEs decrease as the sample size increases, and RMSEs are the lowest when correctly specified model is estimated (“Known Constraint" approach). The unconstrained and *gh*-parsimonious fitted models yield similar RMSE for location and scale parameters of the components. Meanwhile, for mixing proportions, *gh*-parsimonious approach resulted in higher RMSE as compared to unconstrained Tukey’s *g*- &-*h* mixtures across all sample sizes. The highest RMSE of mixing proportions with *gh*-parsimonious approach correspond to the simulated mixtures with 3 or 4 non-zero *g* and *h* parameters. These simulated mixtures are either the full unconstrained models or the models closest in complexity to the full mixture. Also notable, the RMSE of location and scale parameter estimates was higher for the first as compared to the second component due to the larger proportion of observations generated from the second component.

**Fig. 5:**
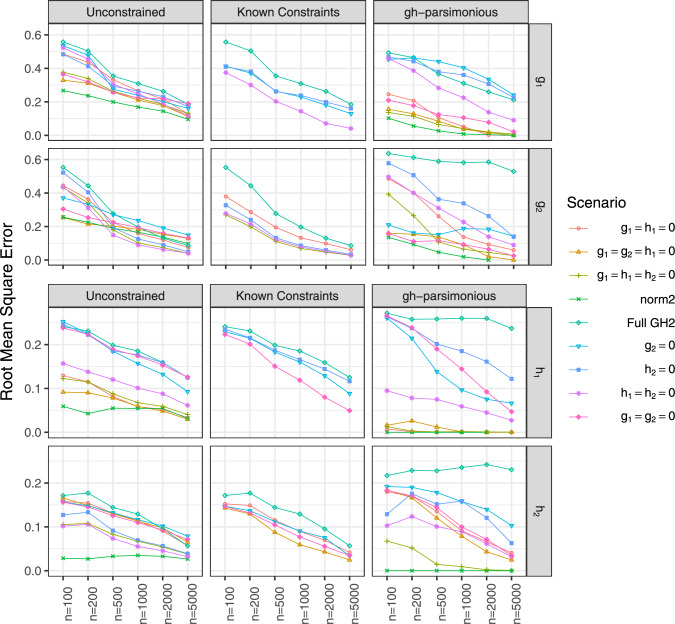
Simulation results, RMSE of the estimates of skewness *g* and kurtosis *h*

The RMSE for *g* and *h* parameters estimates are shown in Figs. 5. For *g* and *h* parameters equal to zero in the true simulated model, the estimates are not computed using “Known Constraint" approach. For *g* and *h* parameters, the RMSE also reduces as the sample size increase, but the rate of decrease is slower as compared to RMSE of location and scale parameters and mixing proportions. The “Known Constraint" approach performs the best estimating *g* and *h* parameters, while *gh*-parsimonious models yield highest RMSE for simulated mixtures with 3 or 4 non-zero *g* and *h* parameters.

### Comparison with skew-normal and skew-*t* mixtures

**Fig. 6:**
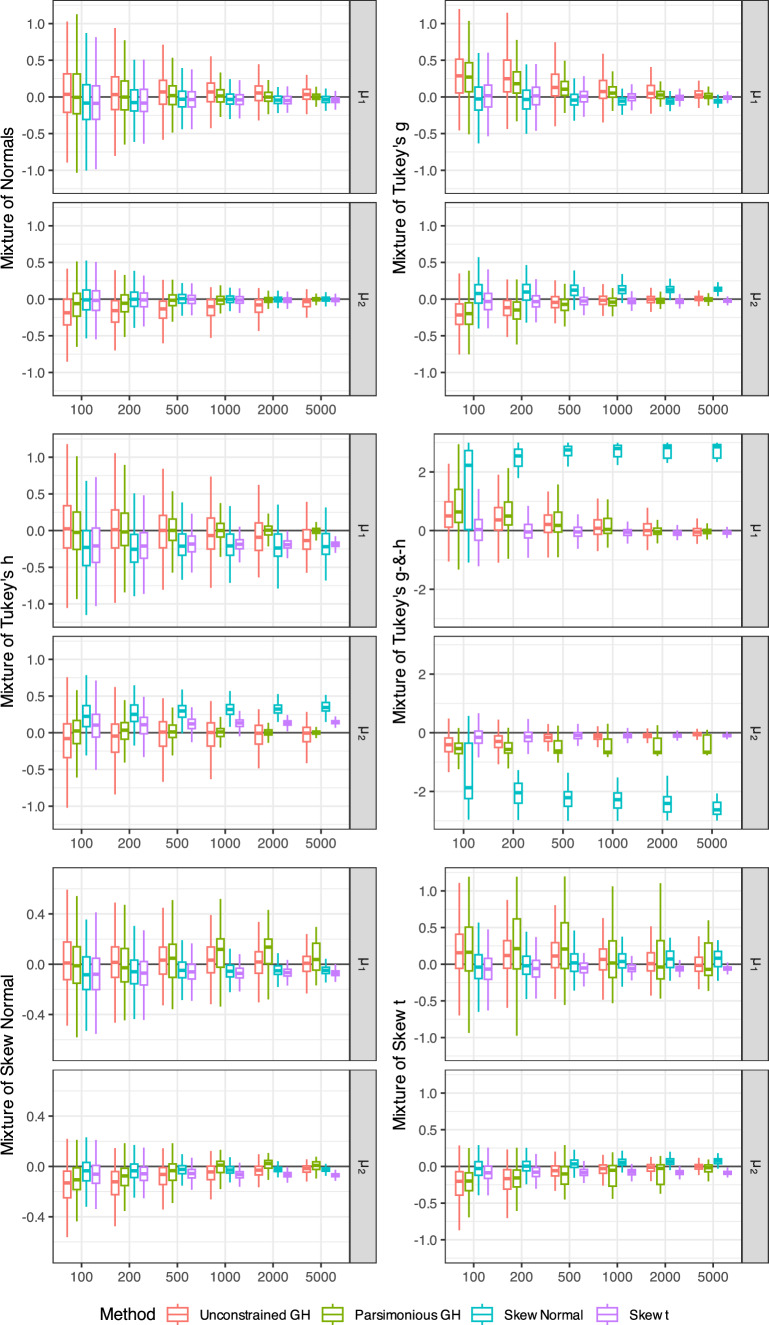
Bias in estimated component means ($\mu _{1}$ and $\mu _{2}$) in simulated the mixtures of normal, skew-normal, skew-*t*, and Tukey’s *g*- &-*h* distributions

Figure 6 shows the bias of estimating the means of the fitted mixtures of skew-normal, skew-*t*, and Tukey’s *g*- &-*h* distributions. The data were simulated using the mixtures of normal, skew-normal, skew-*t*, and Tukey’s *g*- &-*h* distributions, as specified in *y*-axis labels in Fig. 6. The component means of Tukey’s *g*- &-*h*, skew-normal, and skew-*t* distributions were derived from the corresponding moments in Appendix D.

As expected, the smallest bias was observed for the fitted models that were correctly specified. For the simulated mixtures of normal, none of the fitted models was correctly specified, but mixtures of skew-normal, skew-*t*, and parsimonious mixture of Tukey’s *g*- &-*h* distributions performed well approximating normal mixtures when sample size was sufficiently large. Notably, full mixtures of Tukey’s *g*- &-*h* distributions yielded smaller mean bias than mixtures of skew-*t* distributions when the true model was the mixture of skew-normal distributions. Similarly, full mixtures of Tukey’s *g*- &-*h* distributions yielded smaller mean bias than mixtures of skew-normal distributions when the true model was the mixture of skew-*t* distributions. Thus, unconstrained and/or parsimonious Tukey’s *g*- &-*h* mixtures may be expected to provide more robust estimates of the component means if the model is misspecified. Meanwhile, there were no notable differences between estimations methods in terms of goodness of fit as measured by the Cramér-von Mises distance between fitted and empirical distribution (results not shown).

**Fig. 7:**
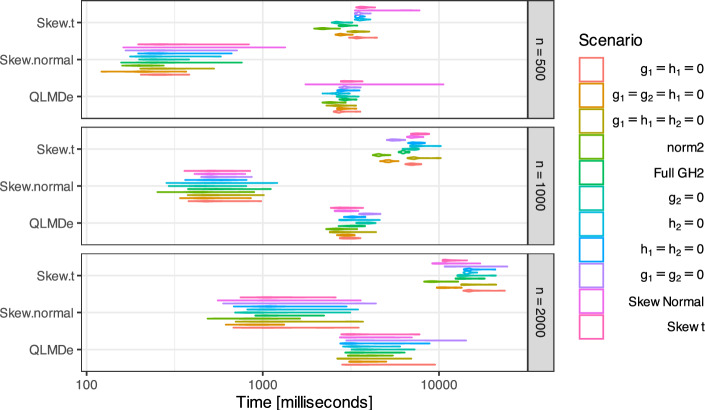
Time cost of QuantileGH::QLMDe vs. mixsmsn::smsn.mix (with options family = "Skew.normal"+ or family = "Skew.t"), based on one random sample generated at $n=500$, 1000, 2000 from each scenario in Fig. 3 and 40 evaluations

Figure 7 compares the running time of function QuantileGH::QLMDe vs. function mixsmsn::smsn. mix (with options family = "Skew.normal" or family = "Skew.t"), with the help of R packages microbenchmark (Mersmann 2023) and parallel. One random sample was generated at each sample size of $n=500$, 1000, 2000 and from each scenario in Fig. 3. Fourty (40) evaluations of each R function were performed using R 4.3.1 on a Mac mini with Apple M1 chip.

The running time for computing unconstrained QLMD estimator was around 5 s for $n=500$ and $n=1000$, but increased to 5–8 s for $n=2000$. The time cost of skew-normal estimator was always lower than for the unconstrained QLMD estimator. Meanwhile is time cost of skew-*t* estimator was either similar (for $n=500$) or higher (for $n=1000$ and $n=2000$) as compared to the time cost of QLMD estimator. Note that the number of parameters in Tukey’s *g*- &-*h* mixtures estimated using QLMD estimator is the same as the number of parameters in skew-*t* mixtures and larger than the number of parameters in skew-normal mixtures.

## Application

### Breast cancer data

We used the Tukey’s *g*- &-*h* mixture model to analyze Cyclin D1 expression levels in cancer cells of treatment-naive breast cancer patients. Cyclin D1 is a protein that belongs to the cyclin family. Cyclins function as regulators of cyclin-dependent kinase. Cyclin D1 over-expression is associated with poor prognosis in estrogen receptor-positive (ER+) breast cancer (Stendahl et al. 2004; Lundberg et al. 2019).

The study population included 690 ER+ patients that had outcome data available, did not have distant metastases at the time of surgery, and had Cyclin D1 CSI expression levels for at least 100 cancer cells in the TMA core IF-IHC image. The progression-free survival (PFS) was defined as the time from diagnosis to the evidence of local, regional or distant recurrence or death from the disease. The additional covariates available included age, race (white vs. non-white), ER status, HER2 status, histologic grade, stage, node status, tumor size ($<2$cm, 2cm-5cm, $>5$cm), and indicator variables for chemotherapy, radiation therapy, and hormone therapy. Patients were classified as “non-compliant" with hormone treatment if it was recommended but patients did not receive this treatment. Ninety three (93) progression events were observed in this study population, with a median follow-up of 89 months (range: 2–238 months).

### Modeling Cyclin D1 distributions as *g*- &-*h* mixtures

The log-transformed Cyclin D1 expression levels in cancer cells, from each patient tissue core, were modeled using three versions of the 2-component Tukey’s *g*- &-*h* mixture,

- unconstrained with all possible 9 parameters;
- *gh*-parsimonious as described in Sect. 3.1
- optimal *gh*-parsimonious with $K_\text {max}=3$, as described in Sect. 3.2

The 2-component mixtures were used because for IF-IHC technology, it is plausible to assume that the CSI distribution is a mixture of a lower component representing the background noise and an upper component representing the actual signal. Mixtures with up to 3 components were considered to allow a possibility that the actual signal distribution is not unimodal.

**Fig. 8:**
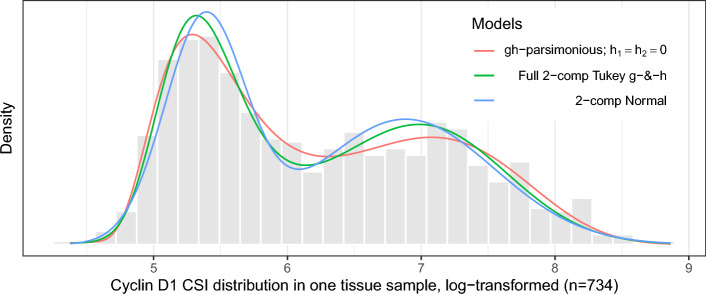
Example of Tukey’s *g*- &-*h* and normal mixtures fitted to one sample tissue

Figure 8 shows an example of the resulting fitted mixtures along with the fitted 2-component normal mixture and kernel density estimator. The estimate of $A_2$ in fitted normal mixture is potentially downward biased as compared to $A_2$ estimate in fitted Tukey’s *g*- &-*h* mixture.

In order to capture the signal in the subpopulations of cells with highest Cyclin D1 expression, we defined $A_\text {max}=\text {max}(A_1,A_2,A_3)$, if fitted distribution is a 3-component Tukey’s *g*- &-*h* mixture, $A_\text {max}=\text {max}(A_1,A_2)$, if fitted distribution is a 2-component Tukey’s *g*- &-*h* mixture, and $A_\text {max}=A_1$, if fitted distribution is a 1-component Tukey’s *g*- &-*h* mixture. The estimated patient-specific Cyclin D1 $A_\text {max}$ values were then evaluated as dichotomized predictor of PFS using a Cox proportional hazard model with bootstrap-based optimism correction procedure (Harrell et al. 1996; Steyerberg 2009). For multivariable Cox model, multiple imputations were used for missing values in clinicopathologic covariates for some patients (0.6% to 10% for all covariates). Forty (40) imputed datasets were created using the Multivariate Imputation by Chained Equations (MICE) algorithm with Fully Conditional Specification (van Stef and Groothuis-Oudshoorn 2011). The bootstrap-based optimism correction was applied to each imputed data set. Then results for all imputed data sets were averaged using the Rubin’s rule (Rubin 2004).

The following steps were performed for the bootstrap optimism correction. First, 200 bootstrap samples were drawn with replacement from each imputed data set with all patients. In each bootstrap sample, the survival tree model (R package rpart) was used to establish an objective data-driven optimal cutpoint for dichotomizing $A_\text {max}$. The cutpoint from the current bootstrap sample was used to compute the log hazard ratio for the univariate or multivariate Cox model in the current bootstrap sample (“Bootstrap performance") and in the main sample (“Test performance"). The optimism in hazard ratio estimation was computed as the difference between log hazard ratio for “Bootstrap performance" and for “Test performance". The optimism estimate was computed as the median optimism over 200 bootstrap samples. The cutpoint for dichotomizing $A_\text {max}$ was also established in each imputed data set and its “apparent performance" was computed as the log hazard ratio for dichotomized $A_\text {max}$ in the corresponding Cox model. Finally, the optimism-corrected performance estimates were computed by subtracting the optimism estimates from the “apparent performance" estimates (Steyerberg 2009).

**Table 2:** Bias-adjusted hazard ratios (HR) with 95% confidence limits for dichotomized $A_\text {max}$ as a predictor of PFS in univariate Cox models

Model for CSI distributions	HR	95%LCL	95%UCL	*P*
Unconstrained 2-comp Tukey’s *g*- &-*h* mixture	1.41	0.89	2.22	0.146
*gh*-parsimonious 2-comp Tukey’s *g*- &-*h* mixture	1.74	1.02	2.99	0.047
Optimal *gh*-parsimonious Tukey’s *g*- &-*h* mixture	1.78	1.18	2.69	0.008

Table 2 shows the bias-adjusted results from the univariate Cox models with dichotomized $A_\text {max}$ as a predictor. Noticeably, the hazards ratio for dichotomized $A_\text {max}$ is the highest based on fitted *gh*-parsimonious Tukey’s *g*- &-*h* mixture with the optimal number of components and the lowest and non-significant for $A_\text {max}=A_2$ in full Tukey’s *g*- &-*h* mixture. The $A_\text {max}=A_2$ in *gh*-parsimonious 2-comp Tukey’s *g*- &-*h* mixture produced slightly lower hazards ratio as compared to the parsimonious Tukey’s *g*- &-*h* mixture. Furthermore, in bias-adjusted multivariate Cox models, only dichotomized $A_\text {max}$ from the optimal *gh*-parsimonious Tukey’s *g*- &-*h* mixture yielded significant predictor of PFS (hazards ratio 1.68, 95%CI: 1.09$-$2.59, p=0.020). The effect of Cyclin D1 in multivariate Cox models is reduced as compared to the univariate Cox models due to association between Cyclin D1 over-expression and known risk factors such as larger tumor size and higher histologic grade (He et al. 2014).

## Discussion

We developed statistical methods and software tools (R package QuantileGH) for modeling complex and possibly multi-modal distributions as finite Tukey’s *g*- &-*h* mixture. This approach allows accommodating mixtures that include components with variable degree of skewness and/or kurtosis. Our simulations assume 2-component mixtures to facilitate computations, but our methodology and R package allow fitting more than 2 components. We used 3-component mixtures for our application, but we have not evaluated the functionality of our package for fitting Tukey’s *g*- &-*h* mixture with four or more components because the number of parameters becomes excessive (up to 19 for four components). Also, it is not clear whether such mixtures of four or more components have interpretational advantages over the standard kernel density estimation.

We have developed a model selection algorithm that allows reducing the number of parameters in the mixture if the data does not support the need of all four Tukey’s *g*- &-*h* parameters for each component. Our simulation results indicate similar performance of full and parsimonious 2-component Tukey’s *g*- &-*h* mixture in terms of RMSE and mean bias, but the reduced model is usually desired for interpretation and higher efficiency. When applied to Cyclin D1 expression data, our model selection of parsimonious Tukey’s *g*- &-*h* mixture for each subject data provided improved results in the population analysis.

In this work, we considered univariate Tukey’s *g*- &-*h* mixtures, but multivariate extensions may be of interests for modeling multidimensional grossly non-Gaussian distributions. Multivariate finite mixtures have been previously considered, for example, with components modeled as multivariate skew-normal distributions (Cabral et al. 2012) or multivariate skew-*t* distributions (Frühwirth-Schnatter and Pyne 2010). As a fully flexible alternative, one can also consider finite mixtures of unspecified multivariate densities as components and estimate the mixture using kernel density estimators (Benaglia et al. 2009). A multivariate version of Tukey’s *g*- &-*h* distribution was developed by Field and Genton (2006) and can be used to define a finite mixture of multivariate Tukey’s *g*- &-*h* distributions.

In conclusion, the proposed finite mixture of Tukey’s *g*- &-*h* distributions model provides a new flexible statistical framework for accommodating multimodal distributions with variably skewed and heavy-tailed components often originating in biomedical applications, but our model could be also useful for econometrics applications.

**Supplementary material**

R-package for QLMD routine: R (R Core Team 2022) package QuantileGH, available at https://CRAN.R-project.org/package=QuantileGH, contains code to perform the QLMD estimation in the article.

## Appendix

### Appendix A: Identifiability of Tukey’s *g*- &-*h* mixtures

Let *T* be any family of distribution functions and $\Upsilon$ be the class of distribution functions of finite mixtures from *T*, i.e., $\Upsilon$ is the convex hull of *T*,

$$\begin{aligned} \Upsilon =&\left\{ \sum ^K_{k=1} w_k F_{\phi _k}(x), K=1, 2, \cdots ;\right. \\ &\left. \qquad \sum ^K_{k=1} w_k = 1, \forall k=1, \cdots , K, w_k>0; F_{\phi _k} \in T \right\} \end{aligned}$$

A class of finite mixture distributions $\Upsilon$ is called *identifiable* if any two elements $\upsilon , \upsilon ' \in \Upsilon$, $\upsilon =\sum ^K_{k=1} w_k F_{\phi _k}(x)$, $\upsilon '=\sum ^{K'}_{k=1} w'_k F_{ \phi '_k}(x)$ are the same, that is $\upsilon \equiv \upsilon '$, if and only if $K=K'$ and the components may be re-ordered so that $w_k=w_k'$ and $F(x, \phi _k)=F(x, \phi '_k)$ for $k=1,\cdots ,K$.

Yakowitz and Spragins (1968) (Yakowitz and Spragins 1968) showed that a necessary and and sufficient condition that $\Upsilon$ be identifiable is that *T* be a linearly independent set over the field of real numbers. The Corollary of this Theorem in (Yakowitz and Spragins 1968) (same as Corollary 3.1.1 in Titterington et al. (1985)) provides that the class $\Upsilon$ of finite mixtures of the family *T* is identifiable if and only if the image of *T* under any vector isomorphism on the span of *T* is linearly independent in the image space.

Two sets of distributions *Q* and *S* are called *disjoint* if their linear spans do not overlap, $\text {span}(Q) \cap \text {span}(S) = \emptyset$ (Browne and McNicholas 2015). The set of distributions *Q* is *identifiable* if the convex hull of *Q* is identifiable, which is equivalent to *Q* being independent (Browne and McNicholas 2015; Yakowitz and Spragins 1968). Further, two sets of distributions *Q* and *S* are called *identifiably disjoint* if each set is *identifiable* (i.e., independent) and the two sets are disjoint, $\text {span}(Q) \cap \text {span}(S) = \emptyset$.

Let $\Upsilon _{Tgh}$ be the class of finite mixture distributions generated by the Tukey’s *g*- &-*h* family $T_{gh}$ with parameter vector $\tau =(A, B, g, h)$, where $B>0$, $h\ge 0$, $A, g, \in \mathbb {R}$,

$$\begin{aligned} T_{gh} = \left\{ F_\tau (x)=P(T_\tau \le x), x \in \mathbb {R} \right\} \end{aligned}$$

We view $\Upsilon _{Tgh}$ as the union of four disjoint distribution classes,

7 $$\begin{aligned} \Upsilon _{T_{gh}} = \Upsilon _{T_{h=0,g=0}} \cup \Upsilon _{T_{h>0, g=0}} \cup \Upsilon _{T_{h=0, g \ne 0}} \cup \Upsilon _{T_{h > 0,g \ne 0}} \end{aligned}$$

where $\Upsilon _{T_{h=0,g=0}}$, $\Upsilon _{T_{h=0,g \ne 0}}$, $\Upsilon _{T_{h>0,g=0}}$ and $\Upsilon _{T_{h>0,g\ne 0}}$ are the classes of finite mixtures of normal distributions, Tukey’s *g*-distributions, Tukey’s *h*-distributions, and Tukey’s *g*- &-*h* distributions, respectively. We will show that each class in equation (7) is identifiable. Then Lemma 1 in Browne and McNicholas (2015) implies that $\Upsilon _{Tgh}$ is identifiable.

Identifiability of finite normal mixtures $\Upsilon _{T_{h=0,g=0}}$ was established by Teicher (1963).

For a pair of fixed parameter values $g_0$ and $h_0 \ge 0$, consider isomorphism that maps each member $N(A,B^2)$ of the normal distributions family into the member of Tukey’s *g*- &-*h* family with parameter vectors $(A,B,g_0,h_0)$. Since the class of finite normal mixtures is identifiable, the main Theorem in (Yakowitz and Spragins 1968) implies that the normal distributions family is linearly independent over the field of real numbers. Hence, the Corollary in (Yakowitz and Spragins 1968) implies that for any pair $g_0,h_0$, the subfamily $T_{g_0,h_0}$ of Tukey’s *g*- &-*h* family with parameters $g_0$ and $h_0 \ge 0$ is linearly independent and the convex hull of $T_{g_0,h_0}$ is identifiable. Moreover, $T_{g_1,h_1}$ and $T_{g_2,h_2}$ are mutually disjoint if $g_1 \ne g_2$ and/or $h_1 \ne h_2$.

Lemma 2 in (Browne and McNicholas 2015) gives that the union of identifiable and mutually disjoint sets $S_{\gamma , \eta _{\gamma }}$ with index parameter $\gamma \in \Gamma$ and other parameters $\eta _{\gamma }$ is identifiable if there exist a total ordering $\prec$ on $\gamma \in \Gamma$.

Each of the three subfamilies of Tukey’s *g*- &-*h* distributions, $T_{g \ne 0,h=0}$ (*g*-distributions), $T_{g=0,h>0}$ (*h*-distributions), and $T_{g \ne 0,h>0}$ can be viewed as a union of identifiable and mutually disjoint subfamilies $T_{g_0,h_0}$ with index parameter $\gamma =(g_0,h_0)$ and other parameters $\eta =(A,B)$ ($\eta$ independent of $\gamma$, as in Corollary 1 in Browne and McNicholas (2015)). We show that a total ordering $\prec$ on $\gamma =(g,h)$ can be defined as $\gamma _1=(g_1,h_1) \prec \gamma _2=(g_2,h_2)$ if $h_1 < h_2$ or if $h_1=h_2$ and $g_1<g_2$.

Consider a linear operator $M_1$ that maps each distribution function $F_\tau (x) \in T_{gh}$ to the integral of $xdF_\tau (x)$ from 0 to the variable upper bound $s>0$. Using the change of variables $u=F_\tau (x)$,

8 $$\begin{aligned} M_1(F_\tau )(s)= \int _{0}^{s} x dF_\tau (x) =\int _{F_\tau (0)}^{F_\tau (s)} t_\tau (u)du \end{aligned}$$

where $t_\tau$ is the quantile function given in (1) and inverse of $F_\tau$, $t_\tau (F_\tau (s))=s$. If $h<1$ then the first moment of $T_\tau$ exists (Martinez and Iglewicz 1984) and integral (8) exists.

Let $M_2$ be the linear operator of differentiation applied to the image of $M_1$,

$$\begin{aligned} M_2 M_1(F_\tau )(s)=&\frac{d}{ds} M_1(F_\tau )(s) = F'_\tau (s) t_\tau (F_\tau (s))\\ =&\frac{s}{ t'_\tau (p)|_{p=F_\tau (s)}} \end{aligned}$$

For $g\ne 0$ and $h>0$, $t_\tau (p)=A+Bg^{-1}(e^{gz_p}-1)e^{hz_p^2/2}$, where $z_p=\Phi ^{-1}_Z(p)$, where $\Phi _Z$ is the distribution function of the standard normal distribution.

Hence, $t'_\tau (p)=\frac{dt}{dz_p}\frac{dz_p}{dp}=Be^{hz_p^2/2} \{e^{gz_p} + g^{-1}(e^{gz_p}-1)hz_p \} \sqrt{2\pi } e^{z_p^2/2}$ and $t'_\tau (p)|_{p=F_\tau (s)}=t'_\tau (p)|_{z_p=s}=B\sqrt{2\pi }e^{(h+1)s^2/2} \{e^{gs} + g^{-1}(e^{gs}-1)hs \}$, so that

$$\begin{aligned} M_2 M_1(F_\tau )(s)= \frac{ (B\sqrt{2\pi })^{-1} s e^{-(h+1)s^2/2}}{e^{gs} + g^{-1}(e^{gs}-1)hs } ,\ \ g\ne 0, h>0 \end{aligned}$$

For $g=0$ and $h>0$, $t_\tau (p)=A+Bz_pe^{hz_p^2/2}$, $t'_\tau (p)=B (1+h z_p^2) e^{hz_p^2/2} \sqrt{2\pi } e^{z_p^2/2}$, so that $t'_\tau (p)|_{p=F_\tau (s)}=B\sqrt{2\pi }(1+h z_p^2)e^{(h+1)s^2/2}$ and

$$\begin{aligned} M_2 M_1(F_\tau )(s)= \frac{ (B\sqrt{2\pi })^{-1} s e^{-(h+1)s^2/2}}{1+h z_p^2}, \ \ g=0, h>0 \end{aligned}$$

For $g\ne 0$ and $h=0$, $t_\tau (p)=A+Bg^{-1}(e^{gz_p}-1)$, $t'_\tau (p)=Be^{gz_p}\sqrt{2\pi } e^{z_p^2/2}$, $t'_\tau (p)|_{p=F_\tau (s)}=t'_\tau (p)|_{z_p=s}=B\sqrt{2\pi } \{e^{gs+s^2/2} \}$, so that

$$\begin{aligned} M_2 M_1(F_\tau )(s)=(B\sqrt{2\pi })^{-1} s e^{-gs-s^2/2} , \ \ g\ne 0, h=0. \end{aligned}$$

For $F_\tau (s)$ in $T_{h=0, g \ne 0}$, let $\phi (s, \tau ) = M_2 M_1(F_\tau )(s)=(B\sqrt{2\pi })^{-1} s e^{-gs-s^2/2}$. Then, for $\tau _1=(A_1, B_1, g_1, 0)$ and $\tau _2=(A_1, B_1, g_2, 0)$, $(g_1,0) \prec (g_2,0)$ is equivalent to $g_1<g_2$ and

$$\begin{aligned} \lim _{s\rightarrow \infty } \frac{\phi (s, \tau _2)}{\phi (s, \tau _1)} =\lim _{s\rightarrow \infty } \frac{(B\sqrt{2\pi })^{-1} s e^{-g_2s-s^2/2}}{(B\sqrt{2\pi })^{-1} s e^{-g_1s-s^2/2}} =0 \end{aligned}$$

For $F_\tau (s)$ in $T_{h>0, g=0}$, let $\phi (s, \tau ) = M_2 M_1(F_\tau )(s)=\frac{ (B\sqrt{2\pi })^{-1} s e^{-(h+1)s^2/2}}{1+h z_p^2}$. Then, for $\tau _1=(A_1, B_1, 0, h_1)$ and $\tau _2=(A_1, B_1, 0, h_2)$, $(0, h_1) \prec (0, h_2)$ is equivalent to $h_1<h_2$ and

$$\begin{aligned} \lim _{s\rightarrow \infty } \frac{\phi (s, \tau _2)}{\phi (s, \tau _1)} = \lim _{s\rightarrow \infty } \frac{(e^{-(h_2+1)s^2/2})(1+h_2 z_p^2)^{-1}}{(e^{-(h_1+1)s^2/2}) (1+h_1 z_p^2)^{-1}} =0 \end{aligned}$$

For $F_\tau (s)$ in $T_{h>0, g\ne 0}$, let $\phi (s, \tau ) = M_2 M_1(F_\tau )(s)= \frac{ (B\sqrt{2\pi })^{-1} s e^{-(h+1)s^2/2}}{e^{gs} + g^{-1}(e^{gs}-1)hs }$. Then, for $\tau _1=(A_1, B_1, g_1, h_1)$ and $\tau _2=(A_1, B_1, g_2, h_2)$, $(g_1, h_1) \prec (g_2, h_2)$ (if $h_1 < h_2$ or if $h_1=h_2$ but $g_1<g_2$) implies

$$\begin{aligned} &  \lim _{s\rightarrow \infty } \frac{\phi (s, \tau _2)}{\phi (s, \tau _1)}\\ &  \qquad =\lim _{s\rightarrow \infty } \frac{e^{(h_1+1)s^2/2}(e^{g_1s} + g_1^{-1}(e^{g_1s}-1)h_1s)}{e^{(h_2+1)s^2/2}(e^{g_2s} + g_2^{-1}(e^{g_2s}-1)h_2s)}=0 \end{aligned}$$

Hence, $(g_1, h_1) \prec (g_2, h_2)$ is the total ordering with properties (i) and (ii) in Theorem 2 of Teicher (1963). Then Lemma 2 in Browne and McNicholas (2015) implies that $\Upsilon _{T_{h>0, g=0}}$, $\Upsilon _{T_{h=0, g \ne 0}}$, and $\Upsilon _{T_{h > 0,g \ne 0}}$ are identifiable, and Lemma 1 in Browne and McNicholas (2015) implies that $\Upsilon _{Tgh}$ (mixtures of any Tukey’s *g*- &-*h* distributions) is identifiable.

### Appendix B: Asymptotic properties of QLMD estimator

It is assumed that *l* pre-specified probabilities $0<p_1<\cdots<p_l<1$ are selected so that

1. $l \ge 5K-1$, where *K* is the number of components in Tukey’s *g*- &-*h* mixture
2. the mapping $\varvec{\theta } \rightarrow \textbf{t}_{\varvec{\theta }} = (t_{\varvec{\theta },p_1}, \cdots , t_{\varvec{\theta },p_l})^t$ is locally injective (that is $\varvec{\theta _1} \ne \varvec{\theta _2}$ implies $\textbf{t}_{\varvec{\theta _1}} \ne \textbf{t}_{\varvec{\theta _2}}$) in some neighborhood of the true parameter vector $\varvec{\theta _0}$
3. the $l \times (5K-1)$ Jacobian matrix $\textbf{J}_{\textbf{t}_{\varvec{\theta }_0}}$ of the partial derivatives with
  $$\begin{aligned} \left[ \textbf{J}_{\textbf{t}_{\varvec{\theta }_0}}\right] _{ij} = \dfrac{\partial t_{\varvec{\theta },p_i}}{\partial \theta _j}\Bigg |_{\varvec{\theta } = \varvec{\theta }_0}\qquad i = 1,\cdots ,l \ \text {and}\ j = 1,\cdots ,5K-1 \end{aligned}$$
  has column rank $(5K-1)$ at the true parameter vector $\varvec{\theta _0}$.

The proposed estimator $\hat{\varvec{\theta }}_\text {QLMD}$ defined in (5) can be viewed as an indirect estimator (Gourieroux et al. 1993; Gouriéroux et al. 1996) with the auxiliary parameter vector $\textbf{t}_{\varvec{\theta }}$ and implicit binding function $h(\varvec{\theta })=\textbf{t}_{\varvec{\theta }}$ that is defined by the system of *l* equations resulting from substituting pre-specified probabilities $0<p_1<\cdots<p_l<1$ into the distribution function of Tukey *g*- &-*h* mixture given in (3).

Theorem 2 in (Mosteller 1946) provides that sample quantile vector $\textbf{t} = (t_{p_1}, \cdots , t_{p_l})^t$ is consistent for estimating the auxiliary parameter quantile vector $\textbf{t}_{\varvec{\theta }}$ of the finite Tukey’s *g*- &-*h* mixture $\textbf{T}_\theta$ and, as $n\rightarrow \infty$,

9 $$\begin{aligned} \sqrt{n}\left( \textbf{t} - \textbf{t}_{\varvec{\theta }} \right) {\mathop {\rightarrow }\limits ^{D}} \text {N}\left( \textbf{0}, \textbf{V}_{\textbf{t}_{\varvec{\theta }}} \right) \end{aligned}$$

where $\textbf{V}_{\textbf{t}_{\varvec{\theta }}}$ has *ij*-th element

$$\begin{aligned} \left[ \textbf{V}_{\textbf{t}_{\varvec{\theta }}} \right] _{ij} = \dfrac{\min \{p_i,p_j\}\ (1-\max \{p_i,p_j\})}{f_{\textbf{T}_\theta }(t_{\varvec{\theta },p_i}) f_{\textbf{T}_\theta }(t_{\varvec{\theta },p_j})}, \quad i,j = 1,\cdots , l \end{aligned}$$

and $f_{\textbf{T}_\theta }$ is the density of the finite Tukey’s *g*- &-*h* mixture $\textbf{T}_\theta$. Density $f_{\textbf{T}_\theta }$ exists since $\textbf{T}_\theta$ is a mixture of absolutely continuous univariate distributions, but $f_{\textbf{T}_\theta }$ does not have a closed form.

Under assumptions (1)-(3) above and consistency and asymptotic normality of the auxiliary parameter $\textbf{t}_{\varvec{\theta }}$, results in (Gourieroux et al. 1993) and Proposition 4.2 in (Gouriéroux et al. 1996) imply that that the indirect estimator $\hat{\varvec{\theta }}_\text {QLMD}$ is consistent for estimating $\varvec{\theta }$ with the asymptotic covariance matrix given in (6).

The approximate asymptotic covariance matrix of the original parameter vector $\varvec{\phi }$ can be obtained from that of the unconstrained parameter vector $\varvec{\theta }$ (6) using the delta method. Recall that

$$\begin{aligned} {\left\{ \begin{array}{ll} A_k = A_1 + \sum _{j = 2}^{k}e^{d_j} & k = 2,\cdots ,K\\ B_k = e^{b_k} & k = 1,\cdots ,K\\ h_k = e^{\eta _k} & k = 1,\cdots ,K\\ w_1 = \dfrac{1}{1+\sum _{j=2}^{K}e^{\pi _j}} \ \text {and} & \\ w_k = \dfrac{e^{\pi _k}}{1+\sum _{j=2}^{K}e^{\pi _j}} & k = 2,\cdots ,K \end{array}\right. } \end{aligned}$$

Jacobian matrix $\textbf{J}_{\varvec{\phi }}=\partial \varvec{\phi }(\varvec{\theta }) / \partial \varvec{\theta }$ has a block-diagonal structure, with (except for elements of 0 or 1)

$$\begin{aligned} {\left\{ \begin{array}{ll} \dfrac{\partial A_m}{\partial d_n} = & e^{d_n}, \quad m = 2,\cdots ,K \text { and } n = 2,\cdots ,m\\ \dfrac{\partial B_k}{\partial b_k} = & e^{b_k}, \quad k = 1,\cdots ,K\\ \dfrac{\partial h_k}{\partial \eta _k} = & e^{\eta _k}, \quad k = 1,\cdots ,K\\ \dfrac{\partial w_1}{\partial \pi _k} = & -\dfrac{e^{\pi _k}}{\left( 1+\sum _{j=2}^K e^{\pi _j}\right) ^2}, \quad k = 2,\cdots ,K\\ \dfrac{\partial w_m}{\partial \pi _n} = & -\dfrac{e^{\pi _m}\cdot e^{\pi _n}}{\left( 1+\sum _{j=2}^K e^{\pi _j}\right) ^2}, \quad m,n = 2,\cdots ,K \end{array}\right. } \end{aligned}$$

For example, a two-component Tukey’s *g*- &-*h* mixture has a $9\times 10$ Jacobian matrix with diagonal blocks

$$\begin{aligned} {\left\{ \begin{array}{ll} \dfrac{\partial (A_1,A_2)^t}{\partial (A_1,d_2)} = & \begin{pmatrix} 1 & 1 \\ 0 & e^{d_2} \end{pmatrix}\\ \dfrac{\partial (B_1,B_2)^t}{\partial (b_1,b_2)} = & \begin{pmatrix} e^{b_1} & 0 \\ 0 & e^{b_2} \end{pmatrix}\\ \dfrac{\partial (g_1,g_2)^t}{\partial (g_1,g_2)} = & I_2\\ \dfrac{\partial (h_1,h_2)}{\partial (\eta _1,\eta _2)} = & \begin{pmatrix} e^{\eta _1} & 0 \\ 0 & e^{\eta _2} \end{pmatrix}\\ \dfrac{\partial (w_1,w_2)^t}{\partial \pi _2} = & \begin{pmatrix} -\dfrac{e^{\pi _2}}{\left( e^{\pi _1}+e^{\pi _2}\right) ^2} & -\dfrac{e^{\pi _2} e^{\pi _2}}{\left( e^{\pi _1}+e^{\pi _2}\right) ^2} \end{pmatrix} \end{array}\right. } \end{aligned}$$

Since $\hat{\varvec{\theta }}_\text {QLMD}$ is a $\sqrt{n}$-consistent estimator of $\theta$, and the transformation $\varvec{\phi }(\varvec{\theta })$ is differentiable with Jacobian matrix $\textbf{J}_{\varvec{\phi }}$, the delta-method implies that as $n\rightarrow \infty$

$$\begin{aligned}&\sqrt{n}\left( \hat{\varvec{\phi }}_\text {QLMD} - \varvec{\phi }_0\right) \\&\qquad {\mathop {\rightarrow }\limits ^{D}}\text {N}\left( \textbf{0}, \textbf{J}_{\varvec{\phi }}^t\cdot \left( \textbf{J}_{\textbf{t}_{\varvec{\theta }_0}}^t\textbf{J}_{\textbf{t}_{\varvec{\theta }_0}}\right) ^{-1}\textbf{J}_{\textbf{t}_{\varvec{\theta }_0}}^t \textbf{V}_{\textbf{t}_{\varvec{\theta }_0}} \textbf{J}_{\textbf{t}_{\varvec{\theta }_0}}\left( \textbf{J}_{\textbf{t}_{\varvec{\theta }_0}}^t\textbf{J}_{\textbf{t}_{\varvec{\theta }_0}}\right) ^{-1}\cdot \textbf{J}_{\varvec{\phi }}\right) \end{aligned}$$

This asymptotic distribution can be used to perform Wald-type test and calculate the $95\%$ confidence intervals for the original parameter $\varvec{\phi }$. For this purpose, the Jacobian matrix $\textbf{J}_{\textbf{t}_{\varvec{\theta }_0}}$ is approximated numerically, for example, using the R (R Core Team 2022) function $\texttt {numericDeriv}$ and the vector of true quantiles $\textbf{t}_{\varvec{\theta }} = (t_{\varvec{\theta },p_1}, \cdots , t_{\varvec{\theta },p_l})^t$ is approximated numerically using $\textbf{t}^*_{\varvec{\theta }}=(t^*_{\varvec{\theta },p_1}, \cdots , t^*_{\varvec{\theta },p_l})^t$ which is the solution of

$$\begin{aligned} p_i= &  \text {Pr}\left( \textbf{T}_\theta< t^*_{\theta ,p_i}\right) \\= &  \sum _{k=1}^K \dfrac{e^{\pi _k}}{\sum _{j}e^{\pi _j}} \text {Pr}\left( Z < T^{-1}_{A_1,d_k,b_k,g_k,\eta _k}(t^*_{\theta ,p_i})\right) , \\ &  \qquad \quad i=1,\cdots ,l \end{aligned}$$

### Appendix C: Modified letter-value based method

We use the letter-value based method described in Hoaglin (1985) with small modifications designed for mixtures of more than one Tukey *g*- &-*h* distribution. Consider a random sample *T* from Tukey *g*-, *h*-, or *g*- &-*h* distribution. For any $0<p<.5$, denote the $p^{th}$ sample quantile by $t_p$. We refer $t_{.5}-t_p$ as the lower half-spread (LHS) and $t_{1-p}-t_{.5}$ as the as upper half-spread (UHS). The modified letter-based estimates are $\hat{A}=t_{.5}$, $\hat{g}=0$ for *h*-distribution or

$$\begin{aligned} \hat{g} = \text {median}\left\{ -\dfrac{1}{z_{p}}\cdot \ln \dfrac{t_{1-p} - t_{.5}}{t_{.5} - t_{p}};\ \ 0<p<.5\right\} \end{aligned}$$

for either the *g*-distribution or the *g*- &-*h* distribution (Hoaglin 1985, equation (10) and (31)). The letter-value based estimates of *B* and *h* depend on whether the Tukey *g*- &-*h* distribution is constrained (some *g* or *h* parameters are zero).

1. In case of normal distribution (i.e., $g=h=0$, not discussed in Hoaglin (1985)), we let $\hat{B}$ be the median absolute deviation (MAD) of the random sample *T*.
2. In case of Tukey’s *g*-distribution (i.e., $h=0$), we let $\hat{B}$ be the slope estimate of the least squares regression of $t_p-\hat{A}$ on $\hat{g}^{-1}(e^{\hat{g}z_p} - 1)$ with the intercept term constrained to zero. This least squares regression can be performed on LHS where $0<p<.5$, on UHS where $.5<p<1$, or on both LHS and UHS (i.e., full-spread).
  Our modification to use regression with fixed zero intercept is inspired by equations (8a) and (8b) of Hoaglin (1985), but it deviates from using least squares regression of $t_p$ on $\hat{g}^{-1}(e^{\hat{g}z_p} - 1)$ on both LHS and UHS described in Hoaglin (1985).
3. In case of Tukey’s *h*-distribution (i.e., $g=0$), since $A-t_p = t_{1-p}-A = -B z_p e^{hz^2_p/2}$, we can perform any of the three least squares regressions
  $$\begin{aligned} {\left\{ \begin{array}{ll} \ln \dfrac{\hat{A} - t_p}{-z_p} & \text {on}\ z_p^2/2 \text {, on LHS, or}\\ \ln \dfrac{t_{1-p} - \hat{A}}{-z_p} & \text {on}\ z_p^2/2 \text {, on UHS, or}\\ \ln \dfrac{t_{1-p} - t_p}{-2z_p} & \text {on}\ z_p^2/2 \text {, on full-spread} \end{array}\right. } \end{aligned}$$
  and let $(\ln \hat{B}, \hat{h})$ be the estimated intercept and slope parameters. This is given in equations (26a), (26b), (27) and (28) of Hoaglin (1985).
4. In case of Tukey’s *g*- &-*h* distribution, we may add up the equations
  $$\begin{aligned} {\left\{ \begin{array}{ll} A-t_p = -Bg^{-1}(e^{gz_p}-1)e^{hz^2_p/2}\\ t_{1-p}-A = Bg^{-1}(e^{-gz_p}-1)e^{hz^2_p/2} \end{array}\right. } \end{aligned}$$
  to get $t_{1-p}-t_p = Bg^{-1}(e^{-gz_p}-e^{gz_p})e^{hz^2_p/2}$. Therefore, we can perform any of the three least squares regressions
  10 $$\begin{aligned} {\left\{ \begin{array}{ll} \ln \dfrac{-\hat{g}(\hat{A} - t_p)}{e^{\hat{g}z_p} - 1} & \text {on}\ z_p^2/2 \text {, on LHS, or}\\ \ln \dfrac{\hat{g}(t_{1-p} - \hat{A})}{e^{-\hat{g}z_p} - 1} & \text {on}\ z_p^2/2 \text {, on UHS, or}\\ \ln \dfrac{\hat{g}(t_{1-p} - t_p)}{e^{-\hat{g}z_p}-e^{\hat{g}z_p}} & \text {on}\ z_p^2/2 \text {, on full-spread} \end{array}\right. } \end{aligned}$$
  and let $(\ln \hat{B}, \hat{h})$ be the estimated intercept and slope parameters.

The use of least squares regression on UHS is discussed in equation (33) of Hoaglin (1985). A similar least squares regression on LHS could be easily derived. In a vague discussion in the paragraph under equation (33) and before the example, Hoaglin implies two potential least squares regressions on full spread: one has been given in equation (10), the other is to let $\ln \hat{B} = (\ln \hat{B}_\text {LHS}+\ln \hat{B}_\text {UHS})/2$ and $\hat{h} = (\hat{h}_\text {LHS}+\hat{h}_\text {UHS})/2$.

All three options of using LHS, UHS or full-spread, are implemented in our R package. Note that in Cases 3 and 4, the least squares regression may yield a negative slope estimate. In such case, we will use $\hat{h}=0$ instead.

### Appendix D: First and second raw moments of skew-normal, skew-*t* and Tukey’s *g*- &-*h* distributions

The first four central moments, i.e., mean, standard deviation, skewness and kurtosis, of a probability distribution could be easily derived from the first four raw moments.

The first and second raw moments of $T_{(gh)}=(T_{(A,B,g,h)} - A)/B$ are (Hoaglin 1985)

$$\begin{aligned} \text {E}\left( T_{(gh)}\right)= &  {\left\{ \begin{array}{ll} 0 & g=0 \\ \dfrac{e^{g^2/(2(1-h))} - 1}{g\sqrt{1-h}} & g\ne 0,\ 0\le h< 1 \end{array}\right. }\\ \text {E}\left( T^2_{(gh)}\right)= &  {\left\{ \begin{array}{ll} (1-2h)^{-3/2} & g=0,\ 0\le h< \frac{1}{2} \\ \dfrac{1}{g^2\sqrt{1-2h}}& \\ \qquad \sum _{i=0}^2 (-1)^i \left( {\begin{array}{c}2\\ i\end{array}}\right) e^{\frac{[(2-i)g]^2}{2(1-2h)}}&g\ne 0,\ 0\le h < \frac{1}{2} \end{array}\right. } \end{aligned}$$

The first and second raw moments of skew-normal (O’Hagan and Leonard 1976) random variable $X_{\text {sn},(\alpha )}= (X_{\text {sn},(\xi ,\omega ,\alpha )} - \xi )/\omega$ are

$$\begin{aligned} {\left\{ \begin{array}{ll} \text {E}\left( X_{\text {sn},(\alpha )}\right) = & \sqrt{\dfrac{2}{\pi }}\cdot \dfrac{\alpha }{\sqrt{1+\alpha ^2}} \\ \text {E}\left( X^2_{\text {sn},(\alpha )}\right) = & 1 \end{array}\right. } \end{aligned}$$

The first and second raw moments of skew-*t* (Azzalini and Capitanio 2003) random variable $X_{\text {st},(\alpha ,\nu )} = (X_{\text {st},(\xi ,\omega ,\alpha ,\nu )}- \xi )/\omega$ are

$$\begin{aligned} {\left\{ \begin{array}{ll} \text {E}\left( X_{\text {st},(\alpha ,\nu )}\right) = & \sqrt{\dfrac{\nu }{\pi }} \cdot \dfrac{\Gamma (\nu /2 - 1/2)}{\Gamma (\nu /2)} \cdot \dfrac{\alpha }{\sqrt{1+\alpha ^2}}\\ \text {E}\left( X^2_{\text {st},(\alpha ,\nu )}\right) = & \nu /(\nu -2) \end{array}\right. } \end{aligned}$$
